# Leiomyosarcoma of the Oropharynx and Neurogenic Tumors in a Young Patient With Turner's Syndrome

**DOI:** 10.1080/13577140120048610

**Published:** 2001-09

**Authors:** Annarosaria De Chiara, Gaetano Apice, Giustino Silvestro, Simona Losito, Gerardo Botti, Francesco Ionna, Vincenzo De Rosa, Annamaria Borghese, Vito Ninfo

**Affiliations:** 1 Istituto Nazionale dei Tumori di Napoli “G. Pascale” Facoltà di Medicina e Chirurgia di Napoli Federico II Italy; 2 Istituto Nazionale dei Tumori di Napoli “G. Pascale” Facoltà di Medicina e Chirurgia di Padova Italy; 3 Servizio di Anatomia Patologica Istituto dei Tumori G. Pascale Via M. Semmola Napoli 80131 Italy

## Abstract

*Patient:* A case of Turner's syndrome developing a leiomyosarcoma of the oropharynx and metachronous neurogenic tumors (mediastinal ‘ganglioneuroblastoma intermixed’, subcutaneous neurilemoma) is described.

*Discussion:* To our knowledge, this case is the second reported leiomyosarcoma in a patient with Turner's syndrome. Also the site of involvement (palate and oropharynx) is particularly unusual for the already rare leiomyosarcomas in the young age.


*Sarcoma* (2001) 5, 151–155

CASE REPORT

### Leiomyosarcoma of the oropharynx and neurogenic tumors in a young patient with Turner's syndrome

ANNAROSARIA DE CHIARA, GAETANO APICE, GIUSTINO SILVESTRO, SIMONA LOSITO, GERARDO BOTTI, FRANCESCO IONNA, VINCENZO DE ROSA, ANNAMARIA BORGHESE° & VITO NINFO*

*Istituto Nazionale dei Tumori di Napoli "G. Pascale", Facoltà di Medicina e Chirurgia di Napoli Federico II°, Facoltà di Medicina e Chirurgia di Padova**

**Abstract**

*Patient:* A case of Turner's syndrome developing a leiomyosarcoma of the oropharynx and metachronous neurogenic tumors (mediastinal 'ganglioneuroblastoma intermixed', subcutaneous neurilemoma) is described.

*Discussion:* To our knowledge, this case is the second reported leiomyosarcoma in a patient with Turner's syndrome. Also the site of involvement (palate and oropharynx) is particularly unusual for the already rare leiomyosarcomas in the young age.

**Key words:** *Turner's syndrome, adolescents, neurogenic tumors, sarcoma, leiomyosarcoma, oropharynx.*

### Introduction

The occurrence of gonadal tumors in gonadal dysgenesis is a well known phenomenon.[1–3] A higher incidence of extragonadal neoplasms with a preponderance of neurogenic tumors is also reported, suggesting that developmental neural defects, hamartomatous growths, and susceptibility to neurogenic tumors are related to one another as an expression of Turner's phenotype.[4] A few example of soft tissue sarcomas are also reported[5–8] but to our knowledge only one leiomyosarcoma (LMS) has already been described in this setting.[9] On the other hand, leiomyosarcoma is a rare tumor in children and adolescents.[10,11] The visceral ones are often related to genetic[12] or acquired immunodeficiency disorders,[13–15] or immunosuppressive drugs[16] and arise in the hepatobiliary, gastrointestinal, and tracheopulmonary systems. In most cases they were shown to be associated with Epstein-Barr virus (EBV).[17]

We describe a leiomyosarcoma occurring in a patient with Turner's syndrome and metachronous neurogenic tumors (mediastinal 'ganglioneuroblastoma intermixed', subcutaneous neurilemoma). Also the site of involvement (palate and oropharynx) is particularly unusual for the already rare leiomyosarcomas at a young age.

### Clinical history

The patient was diagnosed with Turner's syndrome a few months after birth. For this reason, she had estrogen replacement therapy for the last 7 years. She underwent to thoracotomy because of a mediastinal 'ganglioneuroblastoma intermixed' (Shimada: schwannian stroma-rich) (Fig. 1) when she was 4-year-old. She was treated with radiotherapy and she was well and free of disease for 18 years.

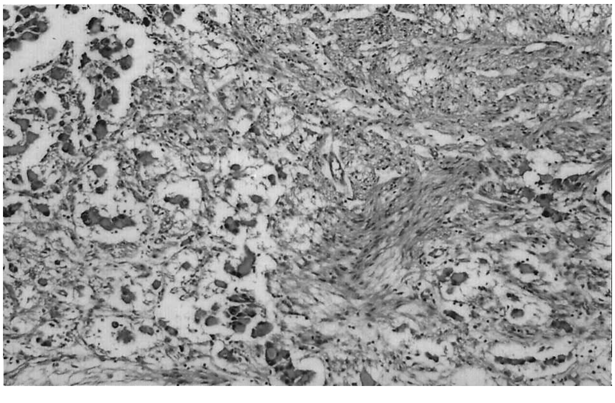

**Fig. 1.** *Mediastinal 'ganglioneuroblastoma intermixed'. In a stromal-rich background, microscopic foci of differentiating neuroblasts and more or less mature ganglion cells are interspersed. (H&E ×10)*

All correspondence should be sent to: Anna De Chiara, Servizio di Anatomia Patologica, Istituto dei Tumori, G. Pascale Via M. Semmola 80131 Napoli Italy. Tel: 039 081 5903359; Fax: 039 081 5461688; E-mail: annadechiara@hotmail.com

1357–714X print/1369–1643 online/01/030151–05 © 2001 Taylor & Francis Ltd
DOI: 10.1080/13577140120071696

At the age of 22, she was referred to our hospital with a huge tumor, more than 10 cm. in largest diameter, infiltrating the whole soft palate, the ventral side of the posterior hard palate, the right tonsillar pillar and deeply into the parapharyngeal space and retromolar trigone on the right side (Fig. 2). An incisional biopsy showed spindle and rounded cells with often vacuolar or clear cell change in the cytoplasm (Fig. 3), strongly positive to muscle specific actin (MSA) (Fig. 4) and to desmin (Fig. 5), with the diagnosis of epithelioid leiomyosarcoma G3. At the same time, two cutaneous nodules (left and right thigh) of 1.5 cm. in largest diameter and a subcutaneous lesion of 2 cm. (in the right axilla) were noted and excised. The cutaneous nodules proved to be sclerotic dermatofibromas with some large atypical mesenchymal cells; the subcutaneous one was a neurilemoma. Serological tests for HBV, HCV, HIV and EBV were negative.

The cytogenetic analysis performed on tissue from the neurilemoma by standard cytogenetic method showed the expected monosomy of chromosome X and chromosome aberrations with a complex karyotype (Fig. 6A). FISH analysis revealed that the most

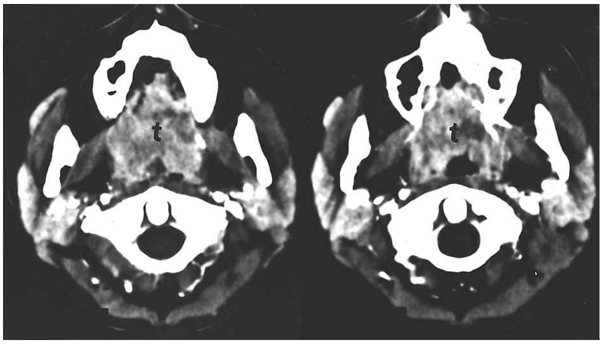

**Fig. 2.** *CT scan sections through the oral cavity show a large mass in the oropharynx infiltrating soft palate with ventral invasion of the posterior edge of the hard palate.*

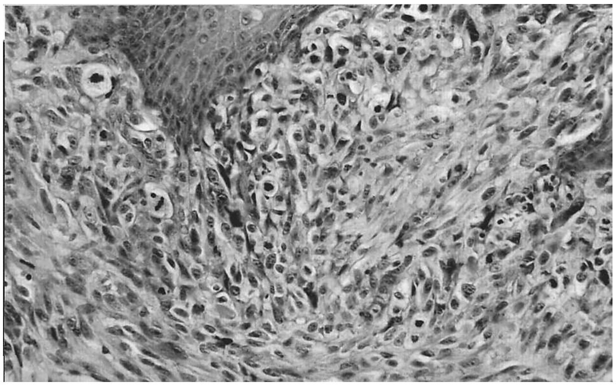

**Fig. 3.** *Epithelioid leiomyosarcoma of the oropharinx. Pleomorphic round to spindle cells with clear or vacuolar cytoplasm and with atypical mitoses are present beneath the oral mucosae. (H&E ×20)*

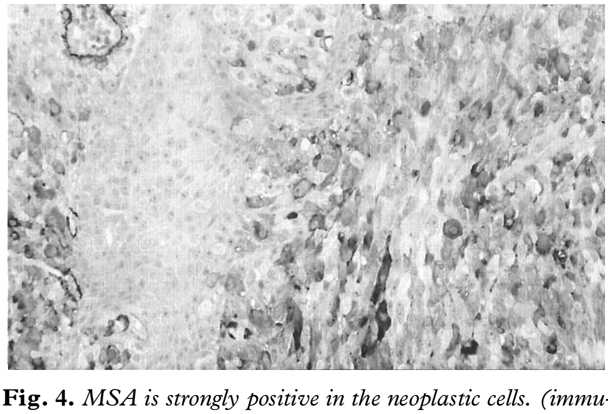

**Fig. 4.** *MSA is strongly positive in the neoplastic cells. (immunoperoxidase ×20)*

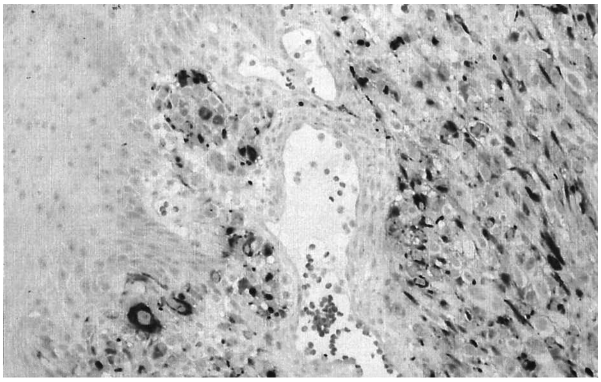

**Fig. 5.** *Desmin is strongly positive in the neoplastic cells. (immunoperoxidase ×20)*

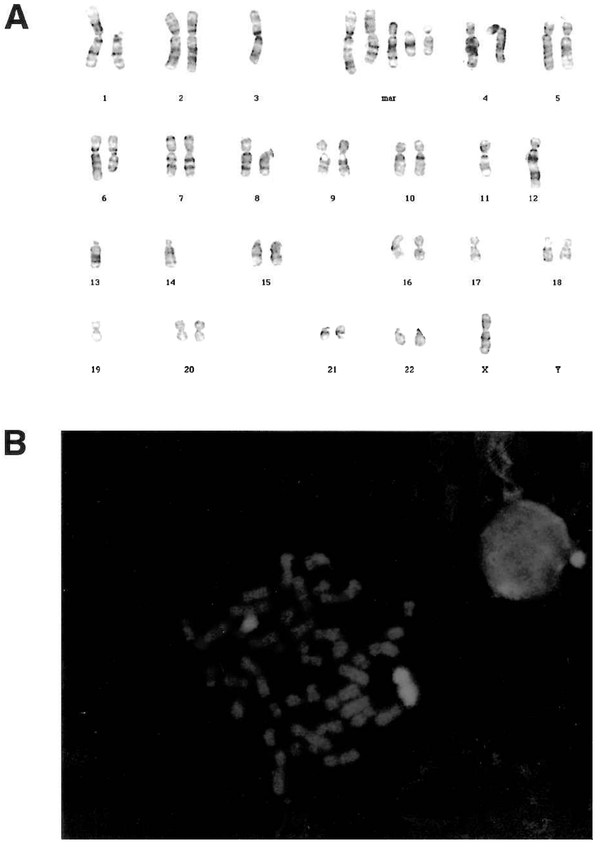

**Fig. 6.** *A) G-banded karyotype of a cell from the neurilemoma shows multiple abnormalities and structural rearrangements such as del(1)(p), del (6)(q22), del (8)(p), der(12)dup(q13–22), loss of a chromosome 13, 14, 19 and X, other unknown rearrangemets defined as markers which probably are derived from chromosome 3, 7, 8, 12 and 18. B) FISH with Chromophobe Multiprobe (Cy 3). Chromosome 6 hybridization: the small spot indicates a rearranged chromosome 6.*

frequent abnormalities were loss of chromosomes 13, 14 and 22, deletions [such as del(1)(p), del (6)(q22)] and rearrangements [such as der(12)dup(q13–22), t(7;9)(q11;q11), t(6;?)] (Fig. 6B). FISH revealed also the presence of part of the Y chromosome translocated on a chromosome of the D group in 10% of the metaphases.

Because of the site and size of the lesion, the patient was treated by chemotherapy (epirubicin 80 mg/m,[2] ifosfamide 7.5 gr/m,[2] DTIC 900 mg/m[2,]), but after two courses she developed a 2.5 cm. right retromandibular lymph node metastases while the oropharyngeal mass was unmodified by CT scan. Then she went to a full course of radiation therapy. The Target Volume included the whole palate, the oropharynx and the lymph nodes of the left neck. A total dose of 66 Gy (2 Gy/fraction) was delivered in 64 days using two lateral portals with 6 MV photons to gross tremor volume (GTV) and bilateral neck lymph nodes, and two opposed antero-posterior fields for supraclavicular nodes for a total dose of 50 Gy. Radiation therapy was stopped on two occasions (a week each time) because of severe laryngeal mucositis. Seventy-five days from the end of radiotherapy, the MRI showed a significant reduction in the tumor mass with fairly good clinical improvement. But a month later, she developed local progression and then lung metastases. She died after a month from respiratory failure.

### Discussion

Leiomyosarcoma is a rare tumor in children and adolescents.[10,11] However, at least some cases reported as LMS, especially those diagnosed in neonates, may represent examples of congenital-infantile myofibromatosis with an excellent prognosis.[18] Some authors also propose that some tumors present in the bronchopulmonary tree, particularly when in endobronchial location, are more often misdiagnosed as LMS while in fact are better regarded as primary bronchopulmonary fibrosarcoma. These histotypes have a relatively good outlook, representing the bronchopulmonary counterpart of the congenital-infantile myofibromatosis of the soft tissue.[19] A recent review of 20 childhood LMSs shows that they have a relatively good prognosis in the majority of the cases and the occurrence of this neoplasm in the oral cavity is exceedingly rare, as already reported.[18,20] While leiomyosarcomas account for about 1% of all soft tissue tumors in childhood (18, also personal experience of one of us, V.N.), they are the second most frequent malignancy in children with acquired immunodeficiency syndrome (AIDS).[15] In this cohort they are often located in unusual sites especially visceral ones such as hepatobiliary, gastrointestinal, and tracheopulmonary systems.[14] Based on the reported cases, EBV seems to be involved in the pathogenesis of these tumors in immunosuppressed patients, while there is no evidence of its presence in the LMS in immunocompetent patients.[17] Thus the oncogenesis of these neoplasms seems to be different in the two subgroups of patients.

Individuals with a history of childhood cancer have been estimated to have 10 to 20 times the lifetime risk of a second cancer compared with age-matched controls. Within the first 20 years after the initial diagnosis, the incidence appears to be on the order of 3% to 12%.[21] In most cases, the second malignancy is related to ionizing radiation or aggressive chemotherapy, especially alkylating agents. The two main criteria to classify a sarcoma as radiation induced are: 1) the patient should be have been irradiated for another malignancy at least 3 years prior to sarcoma diagnosis, and 2) the sarcoma should have developed within the field of radiation. In our case, the patient received radiation for a mediastinal tumor, but the oropharyngeal sarcoma she developed after 18 years was clearly outside of the prior radiation field. She had not received any chemotherapy for the ganglioneuroblastoma. Therefore, we concluded that the second tumor had no links to the therapy.

Alternatively, it is postulated that the occurrence of second cancers may be related to the presence either of an underlying systemic disease (such as von Recklinghausen's neurofibromatosis) or of chromosomal abnormalities shared by multiple organs in which they are tumorigenic such as occurs in some congenital tumors or in Li-Fraumeni syndrome. Thus the simultaneous or sequential appearance of different tumors within an individual may be the clue to their shared genetic etiology. It appears that the majority of hereditary cancer predisposition is attributable to germline mutation in tumor suppressor genes now known to be critically important in growth control of both sporadic and hereditary tumors. Mapping and cloning of the retinoblastoma gene proved Knudson 'two-hits' hypothesis and elucidated the nature of tumor suppressor genes.[22]

The increased risk for malignant transformation within dysgenetic gonads has been documented in numerous reports, most of which occurred in Turner's syndrome with 45,X karyotype or in a phenotypic female with Y chromosome.[2] The most frequent types of tumors in this setting are gonadoblastoma and dysgerminoma.[1,3] Non gonadal malignancies are also reported with a preponderance of melanocytic nevi and peripheral neurogenic neoplasms, both neural crest derivates, among children and young adults.[4] In these cases, it has been postulated that these syndromes may have a mechanism of tumor susceptibility such as occurs in the neurocutaneous disorders, particularly neurofibromatosis type I, in which the NF1 gene is homozygously inactivated in both benign and malignant tumors related to it, and nevoid basal cell carcinoma syndrome (NBCC).[22] So Turner's syndrome may be added to those congenital disorders in which aberrant development of neural-crest derivates predisposes to neoplasia.[4] We have to underline that our patient was affected by Turner's syndrome, cytogenetically diagnosed a few months after birth. She presented a mediastinal 'ganglioneuroblastoma intermixed' (Shimada: schwannian stroma-rich) at the age of 4 years, and later in life a subcutaneous neurilemoma in the right axilla and two sclerotic dermatofibromas in the left and the right thigh.

Some childhood solid tumors occur in close association with hereditary syndromes. For example, soft tissue sarcomas especially rhabdomyosarcoma (RMS), osteosarcoma, brain tumors are frequent in families affected by the Li-Fraumeni syndrome. Survivors of hereditary retinoblastoma, are prone to develop later in life osteosarcomas of bone and soft tissue sarcomas, mainly RMS and fibrosarcoma. In addition, children with neurofibromatosis type I are at increased risk to develop soft tissue malignancies such as malignant peripheral nerve sheath tumors (MPNST), RMS and angiosarcoma.[22] Instead, the occurrence of sarcomas in Turner's syndrome is a very rare event with only few published cases.[4–9] For the endometrial sarcomas, as already postulated for endometrial carcinomas,[23,24] it has been suggested that the risk may be increased by estrogen replacement therapy or endogenous disorders that lead to unopposed estrogenic stimulation of the uterus.[6,7] This mechanism has also been recalled for LMS of the uterus[25] and oral cavity[26] of patients without Turner's syndrome. Experimental studies showed that treatment with testosterone propionate (TP) and dyethylstilbestrol (DES) or TP and estradiol (E2) for 8–9 months causes LMS in vas deferens or uterus of Golden Syrian hamsters at a frequency of 100%. The authors hypothesize that treatment with TP and E2 causes a loss of the protective enzyme hGSTYBX leaving the cells vulnerable to the genotoxic effects of estrogen or estrogenic metabolities.[27]

LMS with clonal abnormalities display highly complex karyotypic changes and extensive heterogeneity, suggesting that the karyotypic profile is more dependent on site of origin than on microscopic features.[28] In our case, the cytogenetic analysis was done on tissue from the subcutaneous neurilemoma that showed a very complex picture with many different rearrangements and deletions. Literature reports that loss of chromosome 22 is frequent in neurilemoma and also the monosomy of chromosome 14 was sometimes observed.[29,30] But the other abnormalities, such as the complex rearrangements involving chromosomes 9 and 12, and the large number of chromosome aberrations resemble more closely the karyotypic changes observed in sarcomas[28] than to the changes described so far in neurilemomas.

To our knowledge, this case is the second reported leiomyosarcoma in a patient with Turner's syndrome, which was also affected by metachronous neurogenic tumors. The site of involvement (palate and oropharynx) is unusual for the already rare leiomyosarcomas in the young age.

### Acknowledgements

We wish to thank Mr. F. Caccavello and Mr. P. Clemente for their technical assistance.

### References

1 Teter J, Boczkowski K. Occurrence of tumors in dysgenetic gonads. *Cancer* 1967; 20:1301–1310.
2 Nam J-H, Chung D-G, Kim Y-M, Kim Y-T, Mok J-E. Endodermal sinus tumor arising from a dysgenetic gonad in a 46,XX female combined with Mullerian agenesis. *Gynecol Oncol* 1994; 55:465–468.
3 Pierga J-Y, Giacchetti S, Vilain E, Extra J-M, Brice P, Espie M, Maragi J-A, Fellous M, Marty M. Dysgerminoma in a pure 45,X Turner syndrome: Report of a case and review of the literature. *Gynecol Oncol* 1994; 55:549–564.
4 Wertelecki W, Fraumeni JF, Mulvihill JJ. Nongonadal neoplasia in Turner's syndrome. *Cancer* 1970; 26:485–488.
5 Males JL, Lain KC. Epithelioid sarcoma in X0/XX Turner's syndrome. *Arch Pathol* 1972; 94:214–216.
6 Mosel A. Endometrial carcino-sarcoma in a young woman with Turner's syndrome. *Geburtshilfe Frauenheilkd* 1979; 39:217–221.
7 Press MF, Scully RE. Endometrial 'sarcomas' complicating ovarian thecoma, polycystic ovarian disease and estrogen therapy. *Gynecol Oncol* 1985; 21:135–154.
8 Miozzo M, Sozzi G, Cariani CT, Di Palma S, Luksch R, Azzarelli A, Pierotti MA, Della Porta G. A synovial sarcoma with t(X;18)(p11,q11) in a patient with Turner's syndrome. *Genes Chrom Cancer* 1992; 4:192–193.
9 Yata Y, Wakabayashi H, Tanaka M, Sawada K, Entani A, Miyamoto A, Andou T, Terasaki T, Yoshio Y, Funaki J. A case of Turner's syndrome complicated with gastric leiomyosarcoma. *Nippon Shokakibyo Gakkai Zasshi* 1994; 91:1328–1332.
10 Yannopoulos K, Purdy Stout A. Smooth muscle tumors in children. *Cancer* 1962; 15/958–971.
11 De Saints Aubain Somerhausen N, Fletcher CDM. Leiomyosarcoma of soft tissue in children. Clinicopathologic analysis of 20 cases. *Am J Surg Pathol* 1999; 23:755–763.
12 Mierau GW, Greffe BS, Weeks DA. Primary leiomyosarcoma of brain in an adolescent with common variable immunodeficiency syndrome. *Ultrastruct Pathol* 1997; 21:301–305.
13 McLoughlin LC, Nord KS, Joshi VV, DiCarlo FJ, Kane MJ. Disseminated leiomyosarcoma in a child with acquired immune deficiency syndrome. *Cancer* 1991; 67:2618–2621.
14 van Hoeven KH, Factor SM, Kress Y, Woodruff JM. Visceral myogenic tumors: A manifestation of HIV infection in children. *Am J Surg Pathol* 1993; 17:1176–1181.
15 Molle ZL, Bonermann P, Desai N, Clarin E, Anderson V, Rabinowitz SS. Endoscopic features of intestinal smooth muscle tumor in a child with AIDS. *Dig Dis SCI* 1999; 44:910–915.
16 Walker D, Gill TJ, Corson JM. Leiomyosarcoma in a renal allograft recipient treated with immunosoppressive drugs. *JAMA* 1971, 215:2084–2086.
17 Rogatsch H, Bonatti H, Menet A, Larcher C, Feichtinger H, Dirnhofer S. Epstein-Barr virus-associated multicentric leiomyosarcoma in an adult patient after heart transplantation. Case report and review of the literature. *Am J Surg Pathol* 2000; 24:614–621.

18 Swanson PE, Wick MR, Dehner LP. Leiomyosarcoma of somatic soft tissues in childhood: An Immunohistochemical analysis of six cases with ultrastructural correlation. *Hum Pathol* 1991; 22:569–577.

19 Pettinato G, Manivel JC, Saldana MJ, Peyser J, Dehner LP. Primary bronchopulmonary fibrosarcoma of childhood and adolescence: Reassement of a low-grade malignancy. *Hum Pathol* 1989; 20:463–471.

20 Das DK, Grover RK, Anand VJ, Mandal AK, Jain S, Jain J, Bhat NC, Chowdhury V. Oral leiomyosarcoma in childhood. Report of a case with fine needle aspiration cytology. *Acta Cytol* 1999; 43:1150–1154.

21 Blatt J, Olshan A, Gula MJ, Dickman PS, Zaranek B. Second malignancies in very-long-term survivors of childhood cancer. *Am J Med* 1992; 93:57–60.

22 DeVita VT Jr., Hellman S, Rosenberg SA. Cancer, Principles & Practice of Oncology. Philadelphia: Lippincott-Raven, 1997.

23 Ostor AG, Fortune DW, Evans JH, Kneale BL: Endometrial carcinoma in gonadal dysgenesis with and without estrogen therapy. *Gynecol Oncol* 1978; 6:316.

24 Mc Carty KS, Barton TK, Peete CH, Creasman WT. Gonadal dysgenesis with adenocarcinoma of the endometrium. An electron microscopic and steroid receptor analysis with a review of the literature. *Cancer* 1978; 42:512–520.

25 Schwartz SM, Weiss NS, Daling JR, Gammon MD, Liff JM, Watt J, Lynch CF, Newcomb PA, Armstrong BK, Thompson WD. Exogenous sex hormone use, correlates of endogenous hormone levels, and the incidence of histologic types of sarcoma of the uterus. *Cancer* 1996; 77:717–724.

26 Goldschmidt PR, Goldschmidt JD, Lieblich SE, Eisenberg E. Leiomyosarcomas presenting as a mandibular gingival swelling: a case report. *J Periodontol* 1999; 70:84–89.

27 Hudson CE, Kelly MM, Schwartz DA, Schofield DA, DeHaven JE, Schulte BA, Norris JS. Glutatione S-transferase in hormonal carcinogenesis. *Chem Biol Interact* 1998; 111–112:343–350.

28 Mandhal N, Fletcher CD, Dal Cin P, De Wever I, Mertens F, Mitelman F, Rosai J, Rydholm A, Sciot R, Tallini G, Van Den Berghe H, Vanni R, Willen H. Comparative cytogenetic study of spindle cell and pleomorphic leiomyosarcomas of soft tissues: a report from the CHAMP Study Group. *Cancer Genet Cytogenet* 2000; 1:66–73.

29 Bello MJ, De Campos JM, Kusak ME, Vaquero J, Sarasa JL, Pestana A, Rey JA. Clonal chromosome aberrations in neurinomas. *Genes Chromosomes Cancer* 1993; 6:206–211.

30 Couturier J, Delattre O, Kujas M, Philippon J, Peter M, Rouleau G, Aurias A, Thomas G. Assessment of chromosome 22 anomalies in neurinomas by combined karyotype and RFLP analyses. *Cancer Genet Cytogenet* 1990; 45:55–62.
